# Comparison of SB17 and reference ustekinumab in healthy adults: A randomized, double-blind, single-dose, phase I study 

**DOI:** 10.5414/CP204492

**Published:** 2024-01-04

**Authors:** Hansol Jeong, Taeseung Kang, Jiyoon Lee, Seongsik Im

**Affiliations:** Samsung Bioepis Co., Ltd., Incheon, Republic of Korea

**Keywords:** biosimilar, ustekinumab, SB17, pharmacokinetics, immunogenicity

## Abstract

Objective: This study compared the pharmacokinetic (PK) characteristics of SB17 (Samsung Bioepis, Incheon, Republic of Korea), a proposed biosimilar of ustekinumab (UST) against reference UST (Stelara, Janssen Biotech, Horsham, PA, USA). Materials and methods: This double-blind, three-arm, parallel-group, single-dose study randomized 201 healthy adult subjects 1 : 1 : 1 to receive 45 mg of SB17, European Union-sourced UST (EU-UST) or United States of America-sourced UST (US-UST) via subcutaneous (SC) injection. Primary endpoints were area under the concentration-time curve from time zero to infinity (AUC_inf_) and maximum serum concentration (C_max_). Safety, tolerability, and immunogenicity were investigated. Results: All 90% confidence intervals (CIs) for the ratios of AUC_inf_ and C_max_ between groups were within the predefined bioequivalence margin of 0.8 – 1.25. The geometric LSMeans ratios of AUC_inf_ and C_max_ were 0.99 and 0.90 for SB17/EU-UST, 1.01 and 0.94 for SB17/US-UST, and 1.02 and 1.05 for EU-UST/US-UST, respectively. The proportion of subjects with treatment-emergent adverse events (TEAEs) was comparable between SB17, EU-UST, and US-UST (68.7, 58.2, and 65.7%). No deaths, serious adverse events (SAEs), or severe TEAEs were reported. The incidence of subjects testing positive for post-dose anti-drug antibodies (ADAs) was 26.9%, 34.3%, and 34.3% in the SB17, EU-UST, and US-UST groups, respectively. Among the subjects with a positive ADA result at day 99/end of study, 53.8% (SB17 n = 5, EU-UST n = 12, and US-UST n = 11) were positive for neutralizing antibodies (NAbs). Conclusion: This study demonstrated bioequivalence of SB17, EU-UST, and US-UST in terms of PK. Safety, tolerability, and immunogenicity were also comparable between all groups.

ClinicalTrials.gov Identifier: NCT04772274. 

EudraCT Number: 2020-004447-88. 

**What is known about this subject**

- Ustekinumab is an approved biological drug targeting interleukins 12 and 23 for the treatment of chronic inflammatory diseases, including psoriasis and inflammatory bowel diseases.
- Biosimilars are products that are highly similar in terms of quality, efficacy, immunogenicity, and safety aspects, and with no clinically meaningful differences compared to the approved reference biologic.

**What this study adds**

- This phase I study confirms that the ustekinumab biosimilar, SB17, has equivalent pharmacokinetics and comparable immunogenicity, tolerability, and safety profiles as reference ustekinumab in adult healthy subjects.
- The results presented in this study further support conducting a phase III study of SB17 in subjects with psoriasis.

## Introduction 

Ustekinumab (UST) (Stelara, Janssen Biotech, Horsham, PA, USA) is a bioengineered fully human immunoglobulin G1 kappa (IgG1κ) monoclonal antibody that targets the p40 subunit of interleukin 12 and interleukin 23 and inhibits the disease-triggering inflammatory signaling cascade [1]. Phase III clinical studies of UST in patients with psoriasis, psoriatic arthritis, Crohn’s disease, and ulcerative colitis had shown efficacy in reducing disease activity scores and achieving or maintaining remission [2, 3, 4, 5]. Accordingly, UST comprises an alternative steroid-free treatment that improves patient’s quality of life. UST was originally approved for the treatment of moderate to severe plaque psoriasis by the U.S. Food and Drug Administration (FDA) and the European Medicines Agency (EMA) in 2009. Later, UST also was approved for the treatment of several chronic inflammatory diseases including psoriatic arthritis, Crohn’s diseases, and moderate to severe ulcerative colitis [6, 7, 8]. 

Biologic medicines such as monoclonal antibodies (mAbs) are a class of drugs that are produced from biological sources [9]. Biologics are complex in size and structure and require sophisticated manufacturing processes, which result in an inherent microheterogeneity of the active ingredient and make the reproduction of identical copies impossible (unlike small molecule generics). Biosimilars are products that are highly similar to an approved reference biologic in terms of quality, efficacy, and safety and have no clinically meaningful differences compared to the reference product [9, 10]. Following the regulatory authority’s (FDA and EMA) “totality of evidence approach” [11, 12], the approval of a biosimilar requires a stepwise approach to demonstrate the “high similarity” between the biosimilar candidate and the reference biologic. This approach starts with showing that no structural and functional meaningful differences exist, followed by showing head-to-head comparability in in vitro assays that are specific and sensitive to detect potential differences between the biosimilar and the reference product [13]. Further non-clinical studies aim to confirm the comparability of critical quality attributes (CQAs) [9, 14],which are followed by clinical phase I studies to evaluate equivalence of pharmacokinetic (PK) properties and provide first data on clinical safety and immunogenicity [15, 16]. The successful development of biosimilars has the potential to facilitate patient’s access to the biologic treatment by lowering the cost barriers and to reduce the burden to the healthcare system [17, 18]. 

SB17 (Samsung Bioepis, Incheon, Republic of Korea) was developed as a biosimilar to Stelara. A series of analytical and non-clinical assays have been performed regarding the structural, physicochemical, and biological characteristics of SB17, confirming that SB17 is highly similar to the reference UST. The phase I study reported here aimed to compare PK parameters, safety, tolerability, and immunogenicity between SB17 and reference UST (EU- and US-sourced ustekinumab, EU-UST and US-UST) in healthy adult subjects. 

## Materials and methods 

### Study design 

This study was a randomized, double-blind, three-arm, parallel-group, single-dose phase I trial (clinicaltrials.gov: NCT04772274) conducted at one center in France. Eligible subjects were randomized in a 1 : 1 : 1 ratio to receive a single dose of 45 mg of SB17, EU-UST, or US-UST via subcutaneous (SC) injection in the periumbilical area on day 1. Subjects were observed for 99 days (2,352 hours) post-dose. The primary PK endpoints of this study were area under the concentration-time curve (AUC) from time zero to infinity (AUC_inf_) and the maximum serum concentration (C_max_). The secondary PK endpoints included the AUC from time zero to the last quantifiable concentration (AUC_last_), AUC from time zero to 264 hours (AUC_0–264h_), time to reach C_max_ (t_max_), apparent volume of distribution during the terminal phase (V_z_/F), terminal rate constant (λ_z_), terminal half-life (T_1/2_), apparent clearance (CL/F), and percentage of AUC_inf_ due to extrapolation from time of last measurable concentration to infinity (%AUC_extrap_). 

### Study population 

Key inclusion criteria of eligible healthy male or female subjects included an age of 18 – 55 years, body weight of 60.0 – 90.0 kg, and a body mass index (BMI) of 19.0 – 29.9 kg/m^2^. The absence of clinically significant abnormalities was confirmed by the investigator, from results of 12-lead electrocardiogram (ECG), vital signs, and physical examination at screening period and day –1 (baseline). Key exclusion criteria included active or latent tuberculosis, a history of serious infection, previous treatment with UST (Stelara or its biosimilar), confirmed or suspected immunogenicity from previous exposure to a monoclonal antibody or fusion protein, and previous exposure to immunosuppressive or biological agents. 

### Ethics 

The study was conducted in compliance with the Declaration of Helsinki and consistent with the International Council for Harmonization and Good Clinical Practice guidelines (ICH-E6 GCP). The final study protocol was approved by the French Independent Ethics Committee (IEC) and the French National Agency for Medicines and Health Products Safety (ANSM). All subjects signed the agreements on the informed consent form (ICF) prior to enrolment. 

### Pharmacokinetic evaluation 

Blood samples for PK analysis were collected from day 1 at 0 hours (pre-dose) and 12, 24, 48, 72, 120, 168, 216, 264, 312, 384, 504, 672, 1,008, 1,344, 1,680, 2,016, and 2,352 hours post-dose. Subjects were discharged on day 3 with the rest of the study period consisting of outpatient visits. Serum concentrations of SB17 and reference USTs were measured using a validated enzyme-linked immunosorbent assay (ELISA) specific for the detection and quantification of UST. The primary endpoint AUC_inf_ was calculated as AUC_last_ + last observed concentration (C_last_)/λ_z_ by linear-up/log-down trapezoidal rule and C_max_ was obtained directly from the concentration-time data. 

### Safety evaluation 

All adverse events (AEs) and serious AEs (SAEs) that occurred from the time the ICF was signed until the end of study (EOS) (i.e., day 99), including events that occurred prior to the administration of the study drugs, have been recorded. The AEs were coded according to the Medical Dictionary for Regulatory Activities (MedDRA) version 23.1 [19] and listed separately by treatment group including subject number, onset/resolution day, system organ class (SOC), the preferred term (PT), seriousness, severity, causality, outcome, and relation to COVID-19. Other safety assessments included laboratory tests (hamatology, chemistry, urinalysis), vital signs, 12-lead electrocardiogram, physical examination, and injection site assessment. 

### Immunogenicity evaluation 

Blood samples for immunogenicity analysis were collected on day 1 (pre-dose), day 29, day 71, and day 99 to analyze anti-drug antibodies (ADAs) and neutralizing antibodies (NAbs) to UST. ADAs were detected using a qualitative and quasi-quantitative electrochemiluminescence immunoassay (ECLIA). ADA-positive samples were further tested for NAbs using ECLIA. 

### Statistical methods 

The sample size determination was based on an inter-subject coefficient of variation (CV) of 33.9% for AUC_inf_, which was reported for a single 90-mg SC administration in healthy male subjects [20]. With the sample size of 63 in each of the treatment arms (and the total sample size of 189), a parallel study design would have 90% power assuming a true geometric mean ratio of 1.05 to be able to reject both the null hypotheses that 1) the true geometric mean ratio of the test to the reference is less than 0.80 and 2) the true geometric mean ratio of test to the reference is greater than 1.25, where both of these null hypotheses can be rejected simultaneously if the 90% confidence intervals (CIs) for the true geometric mean ratio lies completely between 0.80 and 1.25. Assuming a 5% drop-out rate, an actual total of 201 subjects (67 per treatment arm) were recruited and randomized. 

The following sets were used for the analyses performed in the study: (1) the enrolled set consisted of all subjects who had provided informed consent for this study, (2) the randomized set consisted of all subjects who had received a randomization number, (3) the safety set consisted of all subjects who had received the study drug, (4) the PK analysis set consisted of all subjects in the safety set who had had at least one PK sample analyzed without any major protocol deviation that was likely to have had an impact on PK assessment. 

Comparison between treatment groups in baseline characteristics was performed using the χ^2^-test, F-test, or Fisher’s exact test as appropriate. The PK parameters were calculated based on actual sampling times and non-compartmental analysis methods using Phoenix WinNonlin version 8.1 or higher (Certara, St. Louis, MO, USA). AUC was determined by linear-up/log-down trapezoidal rule. No imputations were done in case of missing parts of a date or a time, or when these fields were completely missing. The statistical analysis of the log-transformed primary endpoints was performed by analysis of variance (ANOVA) model with treatment group as a fixed effect. The difference in the least squares means (LSMeans) between SB17 and EU-UST, between SB17 and US-UST, or between EU-UST and US-UST, and the corresponding 90% CIs were determined. Back transformation provided the ratio of geometric LSMeans and 90% CIs for these ratios. Equivalence for the primary endpoints (AUC_inf_ and C_max_) was determined if the 90% CIs for the ratios of geometric LSMeans of SB17 to EU-UST, SB17 to US-UST, and EU-UST to US-UST, respectively, were within the equivalence margin of 0.8 – 1.25. 

## Results 

### Subject disposition, demographic, and baseline characteristics 

**Figure 1.**
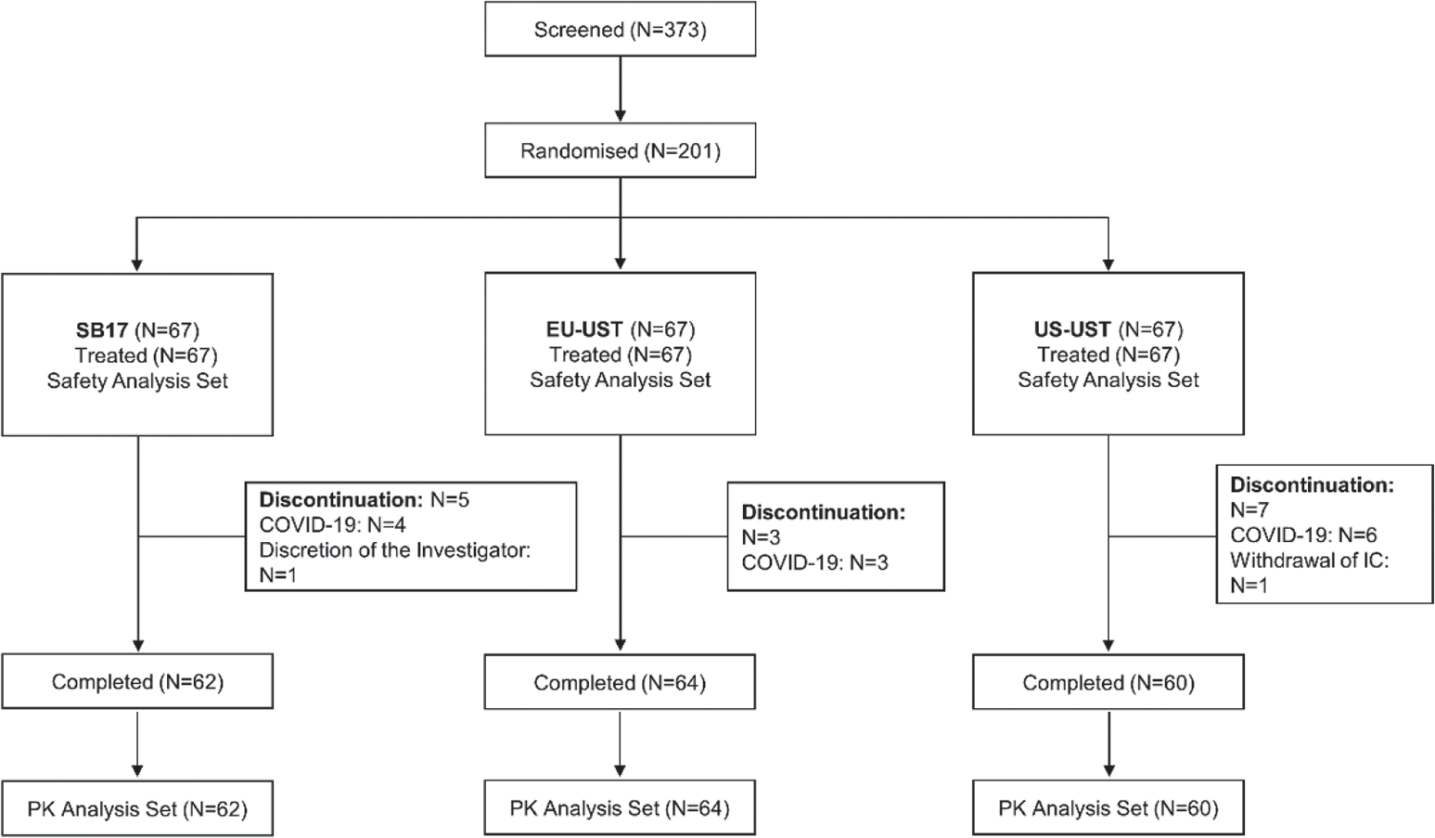
CONSORT diagram of subject disposition. EU-UST = EU-sourced ustekinumab; IC = informed consent; N = number of subjects in each category; PK = pharmacokinetic; SB17 = ustekinumab biosimilar candidate; US-UST = US-sourced ustekinumab.

A total of 373 subjects were screened, of whom 201 subjects were randomized in a 1 : 1 : 1 ratio. Of all subjects who were randomized, 186 completed the study and 15 subjects discontinued the study (Figure 1). Among the 15 discontinued subjects, 13 subjects discontinued due to COVID-19 (categorized as unacceptable toxicity or other AEs), 1 subject withdrew informed consent, and 1 subject was withdrawn at discretion of the investigator (due to pregnancy) (Supplemental Table 1). Demographic and other baseline characteristics were generally comparable among the three treatment groups, with a slightly lower mean value for weight and BMI in the EU-UST group (Table 1). Body weight was identified as a statistically different variable between the treatment groups (p-value = 0.0199) (Table 1). 

### Pharmacokinetic evaluation 

**Figure 2.**
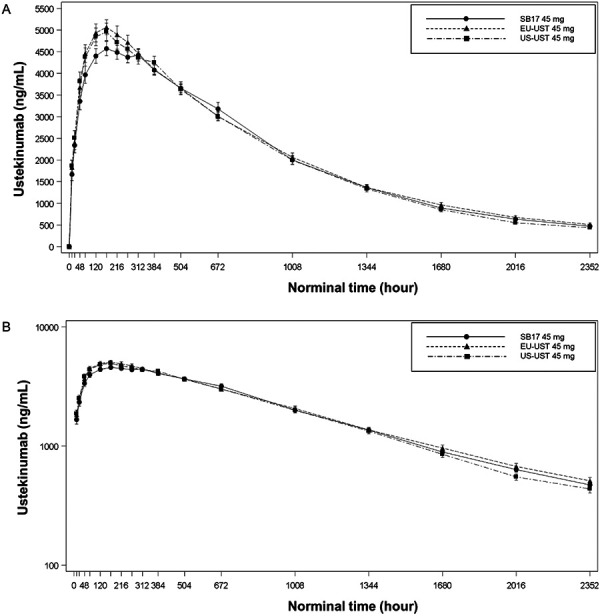
Mean serum concentration-time profiles (Mean ± SD) of USTs. The time dependent UST serum concentrations were comparable between the SB17, EU-UST, and US-UST groups from nominal times zero to 2,352 hours. An increase in the serum concentrations after study drug administration (median t_max_ of 168 hours for all treatment groups) was followed by a slow decrease during the elimination phase. A: Linear scale of UST concentration. B: Semi-logarithmic scale of UST concentration. EU-UST = EU-sourced ustekinumab; SB17 = ustekinumab biosimilar candidate; US-UST = US-sourced ustekinumab.

The mean serum concentration-time profiles for the three treatment-groups were superimposable and comparable between each pair of two groups (Figure 2). A slow increase in the serum concentrations after study drug administration (median t_max_ of 168 hours for all treatment groups) was followed by a slow decrease during the elimination phase. 

The descriptive statistics of the PK parameters are summarized in Table 2. The two primary endpoints were comparable between the SB17, EU-UST, and US-UST treatment groups (mean AUC_inf_: 5,143,600 ng×h/mL, 5,273,000 ng×h/mL, and 5,116,600 ng×h/mL, respectively; mean C_max_: 5,095 ng/mL, 5,689 ng/mL, and 5,420 ng/mL, respectively). Also, all secondary endpoints, including the mean AUC_last_, median t_max_, mean λ_z_ V_z_/F, CL/F, and %AUC_extrap_ values, were comparable across the three treatment groups. In particular, only 2 (1.08%) subjects had higher than 20% values of the %AUC_extrap_, with each of 21.4% and 30.2%. There were 33 PK samples from 33 subjects (16.4%, 1 timepoint/subject) excluded from the analysis of PK parameters due to major protocol deviation (blood samples were centrifuged at low speed; 9 subjects (13.4%) from SB17, 14 subjects (20.9%) from EU-UST, and 10 subjects (14.9%) from US-UST group). However, these subjects were not excluded from any of the analysis sets. 

The geometric LSMeans ratio (90% CI) of AUC_inf_ and C_max_ between the treatment groups were within the predefined equivalence margin of 0.8 – 1.25 (Table 3). Furthermore, weight-adjusted analyses of LSMeans ratios (90% CI) of SB17 vs. EU-UST, SB17 vs. US-UST, and EU-UST vs. US-UST showed that all 90% CIs were within the equivalences margin of 0.8 – 1.25 and contained 1.0 (Table 4). The results of AUC_inf_ were 1.01 (0.92, 1.11), 1.02 (0.94, 1.12), and 1.00 (0.92, 1.10) for SB17 vs. EU-UST, SB17 vs. US-UST, EU-UST vs. US-UST, respectively, while the results of C_max_ were 0.93 (0.85, 1.02), 0.96 (0.88, 1.05), and 1.02 (0.94, 1.12), respectively. 

### Safety evaluation 

A total of 251 treatment-emergent adverse events (TEAEs) were reported in 129 (64.2%) subjects, and all were mild to moderate in severity (Table 5). Among them, 105 TEAEs were reported in 46 (68.7%) subjects of the SB17 group, 79 TEAEs in 39 (58.2%) subjects of the EU-UST group, and 67 TEAEs in 44 (65.7%) subjects of the US-UST group. Overall, the most frequently reported TEAE was headaches (16 TEAEs in 13 (19.4%) subjects from the SB17, 19 TEAEs in 15 (22.4%) subjects from the EU-UST, and 11 TEAEs in 8 (11.9%) subjects from the US-UST group). No deaths, SAEs, or severe TEAEs were observed during the study. There were 14 TEAEs (7.0%) related to COVID-19 reported, and 13 of those led to study discontinuation. One subject with COVID-19 in the US-UST group was diagnosed after consent withdrawal and thus not counted for discontinuation due to TEAEs. 

Laboratory data, vital signs, and ECG parameters did not show any clinically relevant changes that might be considered drug-related over the study duration. 

### Immunogenicity evaluation 

Immunogenicity measurements were available for all subjects, though 10 measurements from 10 subjects (5.0%, 1 timepoint/subject) were excluded from analysis due to major protocol deviation (blood samples were centrifuged at low speed; 2 subjects (3.0%) from SB17, 5 subjects (7.5%) from EU-UST, and 3 subjects (4.5%) from US-UST). However, these subjects were not excluded from any of the analysis sets. 

The overall incidence of subjects with post-dose ADAs to UST was 18/67 (26.9%), 23/67 (34.3%), and 23/67 (34.3%) in the SB17, EU-UST, and US-UST group, respectively (Table 6). Statistical analysis indicated no significant difference in the incidence of post-dose ADAs across the three treatment groups (p-value: 0.4536 for SB17 vs. EU-UST, 0.4536 for SB17 vs. US-UST, and 1.0000 for EU-UST vs. US-UST). Among the subjects with a positive ADA result at day 99/EOS, 28 (53.8%) subjects had a positive result for NAbs (5, 12, and 11 subjects for SB17, EU-UST, and US-UST group, respectively). 

## Discussion 

This single-center, double-blind, three-arm, randomized, parallel-group phase I study was designed to investigate the primary PK parameters, namely the AUC_inf_ and the C_max_, in healthy adult subjects after a single dose (45 mg, SC) of SB17 when compared with the reference UST. The results demonstrated the PK equivalence of SB17 to EU-UST and US-UST, as the 90% CI of LSMeans of AUC_inf_ and C_max_ were within the predefined bioequivalence margin of 0.8 to 1.25. Additional PK parameters, including AUC_last_, AUC_0–264h_, t_max_, V_z_/F, λ_z_, T_1/2_, CL/F, %AUC_extrap_, were also comparable between SB17 and EU-UST, SB17 and US-UST, EU-UST and US-UST. 

The geometric LSMeans ratio (90% CI) of SB17 vs. EU-UST for C_max_ was within the predefined equivalence margin, but it did not include 1.00. Since the demographic and baseline characteristics comparison showed that body weight was statistically different between the groups, an ad-hoc analysis of covariance (ANCOVA) for the primary PK parameters with body weight as a covariate was performed. The 90% CI ranges of the weight-adjusted LSMeans ratios of AUC_inf_ and C_max_ for SB17 vs. EU-UST, SB17 vs. US-UST and EU-UST vs. US-UST were within the predefined equivalence margin of 0.8 – 1.25 and contained 1.00, confirming PK equivalence independent of the differences in body weight. The observation of lower serum UST concentrations in heavier subjects was in line with results from some other studies of UST [6, 21]. Regarding the %AUC_extrap,_ 2 (1.08%) subjects’ %AUC_extrap_ values higher than 20% were considered as the acceptable results. In line with the EMA guidance, as 98.92% subjects having lower than 20% of the %AUC_extrap_, overall reliable estimate of the extent of the exposure was further supported [22]. 

The proportion of subjects who experienced TEAEs was comparable among all three treatment groups, and the laboratory data, vital signs, and 12-lead ECG parameters did not show any clinically relevant changes that might be considered drug-related over the study period. There were no deaths, SAEs, severe TEAEs, and no study drug-related discontinuations due to TEAEs during this study. 

Therapeutic proteins (biologics) can elicit an immune response and consequently the production of ADAs or NAbs (i.e., ADAs against the antigen-binding site of therapeutic mAbs) [23]. The negative influence of ADAs on a drug’s PK can lead to potential loss of efficacy or initiation of drug tolerance [24], and it was reported that ADAs in response to UST treatment had an influence on the PK profiles of UST in patients with psoriasis and psoriatic arthritis [7, 8]. Confirmation of comparable immunogenicity is an important aspect of demonstrating bioequivalence between biosimilars and reference biologics. In this study, post-dose ADA incidence was not statistically significantly different across the three treatment groups. Apart from small numerical differences in post-dose ADA incidence, PK profiles were comparable among the ADA-negative subjects in each group. The subgroup analysis indicated that ADA-negative subjects had consistent PK profiles regarding their primary PK parameters (Supplemental Table 2). The mean values of primary PK parameters for ADA-positive subjects tended to be lower than those for ADA-negative subjects within each group but remained comparable across the groups. 

## Conclusion 

This randomized, double-blind phase I study of UST biosimilar candidate, SB17, and reference UST in healthy adult subjects showed comparable PK parameters, safety, and immunogenicity between the treatment groups, confirming the bioequivalence of 45 mg single SC dose between SB17 and EU- as well as US-sourced UST. Overall, the results further support to conduct a phase III study of SB17 in patients with psoriasis. 

## Acknowledgment 

Medical writing support was provided by SFL Regulatory Affairs & Scientific Communication and funded by Samsung Bioepis. 

MedDRA trademark is registered by ICH. 

## Authors’ contributions 

H.J. and T.K. were responsible for study concepts and design; J.L. and S.I. were responsible for data acquisition; H.J., T.K., and J.L. were responsible for data analysis and interpretation; H.J. and T.K. were responsible for manuscript preparation; all authors were responsible for manuscript review and approval. 

## Funding 

Planning, conduct, and analysis of the study were funded by Samsung Bioepis Co., Ltd., Incheon, Republic of Korea. 

## Conflict of interest 

This study was sponsored by Samsung Bioepis Co., Ltd. 

All authors are employees of Samsung Bioepis Co., Ltd. 

There are no other relationships or activities that could appear to influence the submitted work. 

**Table 1.** Baseline demographics and characteristics.

Characteristics	SB 17 N = 67	EU-UST N = 67	US-UST N = 67	Total N = 201	p-value
Age, years	34.9 (10.75)	33.0 (10.16)	33.4 (10.79)	33.8 (10.55)	0.5604^a^
Gender, n					0.9792^b^
Female	26 (38.8)	25 (37.3)	26 (38.8)	77 (38.3)	
Male	41 (61.2)	42 (62.7)	41 (61.2)	124 (61.7)	
Height, cm	173.6 (8.05)	172.6 (7.05)	172.9 (8.54)	173.0 (7.87)	0.7486^a^
Weight, kg	74.89 (7.396)	71.25 (7.593)	73.42 (7.537)	73.19 (7.621)	0.0199^a^
BMI, kg/m^2^	24.88 (2.256)	23.94 (2.348)	24.62 (2.259)	24.48 (2.311)	0.0520^a^
Race, n					0.8075^c^
White	56 (83.6)	56 (83.6)	58 (86.6)	170 (84.6)	
Black or African American	9 (13.4)	6 (9.0)	6 (9.0)	21 (10.4)	
American Indian or Alaska Native	2 (3.0)	1 (1.5)	1 (1.5)	4 (2.0)	
Native Hawaiian or other Pacific Islander	0 (0.0)	1 (1.5)	0 (0.0)	1 (0.5)	
Other	0 (0.0)	1 (1.5)	0 (0.0)	1 (0.5)	
Multiple	0 (0.0)	2 (3.0)	2 (3.0)	4 (2.0)	
Ethnicity, n					1.0000^c^
Hispanic or Latino	2 (3.0)	2 (3.0)	1 (1.5)	5 (2.5)	
Not Hispanic or Latino	65 (97.0)	65 (97.0)	66 (98.5)	196 (97.5)	

Data presented as mean (SD) or n (%). BMI = body mass index; EU-UST = EU-sourced ustekinumab; N = number of subjects in the randomized set; n = number of subjects within the category; SB17 = ustekinumab biosimilar candidate; SD = standard deviation; US-UST = US-sourced ustekinumab. Percentages were based on the number of subjects in the randomized set. ^a^Comparison between treatment groups was performed using the F-test. ^b^Comparison between treatment groups was performed using the χ^2^-test. ^c^Comparison between treatment groups was performed using the Fisher’s exact test.

**Table 2.** Summary statistics of pharmacokinetic parameters.

PK parameter	Statistics	SB17 N = 62	EU-UST N = 64	US-UST N = 60
AUC_inf_ (ng×h/mL)	Mean (SD)	5,143,600 (1,401,400)	5,273,000 (1,649,100)	5,116,600 (1,526,800)
	Median (Min – Max)	4,969,900 (2,414,000 – 8,884,000)	5,305,400 (2,045,000 – 10,280,000)	4,918,500 (2,118,000 – 10,200,000)
C_max_ (ng/mL)	Mean (SD)	5,095 (1,498)	5,689 (1,877)	5,420 (1,659)
	Median (Min – Max)	5,045 (2,630 – 9,030)	5,480 (2,950 – 13,700)	5,400 (1,590 – 11,100)
AUC_last_ (ng×h/mL)	Mean (SD)	4,721,000 (1,262,300)	4,853,500 (1,431,100)	4,769,900 (1,336,800)
	Median (Min – Max)	4,642,600 (2,302,000 – 8,526,000)	4,993,000 (1,974,000 – 9,786,000)	4,644,100 (1,959,000 – 9,257,000)
AUC_0–264h_ (ng×h/mL)	Mean (SD)	1,044,800 (325,730)	1,148,200 (365,750)	1,117,700 (355,820)
	Median (Min – Max)	1,039,000 (458,500 – 1,780,000)	1,104,500 (565,500 – 2,820,000)	1,105,300 (291,500 – 2,042,000)
t_max_ (h)	Median (Min – Max)	168.000 (48.00 – 672.00)	168.000 (12.00 – 504.00)	168.000 (48.00 – 1,008.00)
V_z_/F (mL)	Mean (SD)	7,561.0 (2,312.5)	7,149.3 (1,744.3)	7,240.6 (2,241.5)
	Median (Min – Max)	7,250.2 (2,817 – 12,910)	6,945.2 (2,825 – 11,560)	6,941.4 (3,207 – 17,980)
λ_z_ (1/h)	Mean (SD)	0.0013891 (0.00084317)	0.0014051 (0.00083473)	0.0013624 (0.00035215)
	Median (Min – Max)	0.0011864 (0.0007264 – 0.005569)	0.0012144 (0.0007386 – 0.007060)	0.0012921 (0.0007192 – 0.002264)
T_1/2_ (h)	Mean (SD)	582.70 (171.00)	563.80 (161.55)	541.07 (134.93)
	Median (Min – Max)	584.24 (124.5 – 954.2)	571.09 (98.2 – 938.4)	536.46 (306.1 – 963.8)
CL/F (mL/h)	Mean (SD)	9.4308 (2.7416)	9.4592 (3.3800)	9.6075 (3.0698)
	Median (Min – Max)	9.0546 (5.065 – 18.64)	8.4819 (4.378 – 22.01)	9.1494 (4.411 – 21.25)
%AUC_extrap_	Mean (SD)	7.91 (5.00)	7.40 (3.73)	6.32 (3.18)
	Median (Min – Max)	6.41 (1.4 – 30.2)	6.69 (1.6 – 18.5)	5.52 (2.2 – 14.9)

AUC_0–264h_ = AUC from time zero to 264 hours; AUC_last_ = AUC from time zero to the last quantifiable concentration; AUC_inf_ = area under the concentration-time curve from time zero to infinity; C_max_ = maximum serum concentration; CL/F = apparent clearance; EU-UST = EU-sourced ustekinumab; Min = minimum; Max = maximum; N = number of subjects for the assessment parameter; PK = pharmacokinetic; SB17 = ustekinumab biosimilar candidate; SD = standard deviation; T_1/2_ = terminal half-life; t_max_ = time to reach C_max_; US-UST = US-sourced ustekinumab; V_z_/F = apparent volume of distribution during the terminal phase; λ_z_ = terminal rate constant; %AUC_extrap_ = percentage of AUC_inf_ due to extrapolation from time of last measurable concentration to infinity. Median and Min-Max range were summarized for t_max_. Samples with low-speed centrifuge issue were excluded from PK parameters calculation. Refer to Pharmacokinetic evaluation of Results section.

**Table 3.** Statistical comparison of primary pharmacokinetic parameters between treatment groups.

PK parameter	Treatment	N	n*	Geo-LSMean
AUC_inf_ (ng×h/mL)	SB17	67	62	4,958,100
	EU-UST	67	64	5,020,000
	US-UST	67	60	4,900,400
C_max_ (ng/mL)	SB17	67	62	4,882
	EU-UST	67	64	5,433
	US-UST	67	60	5,169
PK Parameter	Comparison		Ratio	90% CI of Ratio
AUC_inf_ (ng×h/mL)	SB17/EU-UST		0.99	[0.90, 1.08]
	SB17/US-UST		1.01	[0.93, 1.10]
	EU-UST/US-UST		1.02	[0.93, 1.12]
C_max_ (ng/mL)	SB17/EU-UST		0.90	[0.82, 0.98]
	SB17/US-UST		0.94	[0.86, 1.04]
	EU-UST/US-UST		1.05	[0.96, 1.15]

AUC_inf_ = area under the concentration-time curve from time zero to infinity; C_max_ = maximum serum concentration; CI = confidence interval; N = number of subjects in the PK analysis set; n = number of subjects in the analysis; EU-UST = EU-sourced ustekinumab; Geo-LSMean = geometric least squares mean; PK = pharmacokinetic; SB17 = ustekinumab biosimilar candidate; US-UST = US-sourced ustekinumab. *In total, 15 subjects were excluded from the ANOVA analysis (13 due to COVID-19, 1 due to the discretion of the investigator, and 1 due to withdrawal of informed consent).

**Table 4.** ANCOVA for the primary pharmacokinetic parameters with weight as a covariate.

PK parameter	Treatment	N	n*	Geo-LSMean
AUC_inf_ (ng×h/mL)	SB17	67	62	5,015,600
	EU-UST	67	64	4,964,200
	US-UST	67	60	4,870,100
C_max_ (ng/mL)	SB17	67	62	4,977
	EU-UST	67	64	5,332
	US-UST	67	60	5,119
PK parameter	Comparison		Ratio	90% CI of ratio
AUC_inf_ (ng×h/mL)	SB17/EU-UST		1.01	[0.92, 1.11]
	SB17/US-UST		1.02	[0.94, 1.12]
	EU-UST/US-UST		1.00	[0.92, 1.10]
C_max_ (ng/mL)	SB17/EU-UST		0.93	[0.85, 1.02]
	SB17/US-UST		0.96	[0.88, 1.05]
	EU-UST/US-UST		1.02	[0.94, 1.12]

ANCOVA = analysis of covariance; AUC_inf_ = area under the concentration-time curve from time zero to infinity; C_max_ = maximum serum concentration; CI = confidence interval; EU-UST = EU-sourced ustekinumab; Geo-LSMean = geometric least squares mean; N = number of subjects in the PK analysis set; n = number of subjects in the analysis; PK = pharmacokinetic; SB17 = ustekinumab biosimilar candidate; US-UST = US-sourced ustekinumab. *In total, 15 subjects were excluded from the ANOVA analysis (13 due to COVID-19, 1 due to the discretion of the investigator, and 1 due to withdrawal of informed consent).

**Table 5.** Summary of adverse events.

Number of subjects experiencing	SB17 N = 67		EU-UST N = 67		US-UST N = 67		Total N = 201	
	n (%)	E	n (%)	E	n (%)	E	n (%)	E
Any AEs	46 (68.7)	109	39 (58.2)	80	45 (67.2)	71	130 (64.7)	260
Any TEAEs	46 (68.7)	105	39 (58.2)	79	44 (65.7)	67	129 (64.2)	251
TEAE severity								
Mild	15 (22.4)	53	14 (20.9)	39	29 (43.3)	42	58 (28.9)	134
Moderate	31 (46.3)	52	25 (37.3)	40	15 (22.4)	25	71 (35.3)	117
Severe	0 (0.0)	0	0 (0.0)	0	0 (0.0)	0	0 (0.0)	0
TEAE causality								
Not related	29 (43.3)	78	24 (35.8)	58	31 (46.3)	53	84 (41.8)	189
Related	17 (25.4)	27	15 (22.4)	21	13 (19.4)	14	45 (22.4)	62
Any SAEs	0 (0.0)	0	0 (0.0)	0	0 (0.0)	0	0 (0.0)	0
TEAEs related to COVID-19	4 (6.0)	4	3 (4.5)	3	7 (10.4)	7	14 (7.0)	14
TEAEs leading to study discontinuation	4 (6.0)	4	3 (4.5)	3	6 (9.0)	6	13 (6.5)	13
Deaths	0 (0.0)	0	0 (0.0)	0	0 (0.0)	0	0 (0.0)	0
Any other TEAEs with incidence > 5% of subjects	35 (52.2)	53	30 (44.8)	45	31 (46.3)	40	96 (47.8)	138
Nasopharyngitis	8 (11.9)	9	10 (14.9)	11	7 (10.4)	7	25 (12.4)	27
Gastroenteritis	5 (7.5)	5	0 (0.0)	0	1 (1.5)	1	6 (3.0)	6
Rhinitis	5 (7.5)	5	2 (3.0)	3	3 (4.5)	3	10 (5.0)	11
COVID-19	4 (6.0)	4	3 (4.5)	3	7 (10.4)	7	14 (7.0)	14
Pharyngitis	4 (6.0)	4	1 (1.5)	1	1 (1.5)	1	6 (3.0)	6
Blood creatine phosphokinase increased	2 (3.0)	2	0 (0.0)	0	6 (9.0)	6	8 (4.0)	8
Back pain	4 (6.0)	4	6 (9.0)	6	3 (4.5)	3	13 (6.5)	13
Headache	13 (19.4)	16	15 (22.4)	19	8 (11.9)	11	36 (17.9)	46
Oropharyngeal pain	4 (6.0)	4	2 (3.0)	2	1 (1.5)	1	7 (3.5)	7

AE = adverse event; E = frequency of adverse events; EU-UST = EU-sourced ustekinumab; N = number of subjects randomized in the safety set; n = number of subjects reported AEs; SAE = serious adverse event; SB17 = ustekinumab biosimilar candidate; TEAE = treatment-emergent adverse event; US-UST = US-sourced ustekinumab. Percentages were based on N. If a subject had multiple events with different severity (or causality), then the subject was counted only once at the worst severity (or causality (i.e., related)) for the number of subjects (n).

**Table 6.** Incidence of anti-drug antibodies and neutralizing antibodies to ustekinumab.

Parameter		SB17 N = 67		EU-UST N = 67		US-UST N = 67		Total N = 201	
Timepoint	Assessment	n/n’	(%)	n/n’	(%)	n/n’	(%)	n/n’	(%)
ADAs									
Day 1 pre-dose (baseline)	Positive	2/67	(3.0)	4/67	(6.0)	2/67	(3.0)	8/201	(4.0)
Day 29	Positive	10/66	(15.2)	6/65	(9.2)	6/65	(9.2)	22/196	(11.2)
Day 71	Positive	9/63	(14.3)	12/62	(19.4)	14/62	(22.6)	35/187	(18.7)
Day 99	Positive	14/62	(22.6)	19/62	(30.6)	19/60	(31.7)	52/184	(28.3)
Post-dose (all time)	Positive	18/67	(26.9)	23/67	(34.3)	23/67	(34.3)	64/201	(31.8)
NAbs									
Day 1 pre-dose (baseline)	Positive	0/2	(0.0)	1/4	(25.0)	0/2	(0.0)	1/8	(12.5)
Day 29	Positive	6/10	(60.0)	2/6	(33.3)	2/6	(33.3)	10/22	(45.5)
Day 71	Positive	5/9	(55.6)	10/12	(83.3)	11/14	(78.6)	26/35	(74.3)
Day 99	Positive	5/14	(35.7)	12/19	(63.2)	11/19	(57.9)	28/52	(53.8)

ADAs = anti-drug antibodies; EU-UST = EU-sourced ustekinumab; N = number of subjects in the safety analysis set. n = number of subjects within assessment category; n’ = number of subjects with available assessment at each timepoint; NAbs = neutralizing antibodies; SB17 = ustekinumab biosimilar candidate; US-UST = US-sourced ustekinumab. Percentages were based on n’. Post-dose ADAs was defined as Positive if subjects had at least one ADA-positive on post-baseline and Negative if subjects had no ADAs positive on post-baseline. NAbs results were summarized for ADA positive subjects only. Samples with low-speed centrifuge issue were excluded from analysis of post-dose ADA status. Refer to Immunogenicity evaluation of Results section.

## Supplemental material

Supplemental material Supplemental Tables 1 and 2
