# Supplementary material for: Comparison of SB17 and reference ustekinumab in healthy adults: A randomized, double-blind, single-dose, phase I study
Source: Int J Clin Pharmacol Ther. 2024 Jan 4;62(5):231–40. doi: 10.5414/CP204492 (PMC11036876; doi:10.5414/CP204492)
Supplement: Supplemental material [file intjclinpharmacol-62-231-S01.pdf]

Supplementary table 1. Subject disposition

	SB 17<br>N=67	EU-UST<br>N=67	US-UST<br>N=67	Total<br>N=201
Subjects	n (%)	n (%)	n (%)	n (%)
Screened	373			373
Screening failures	n.a.	n.a.	n.a.	172
Reason for screening failures				
Consent withdrawal	n.a.	n.a.	n.a.	21 (12.2)
Inclusion/exclusion criteria	n.a.	n.a.	n.a.	145 (84.3)
Other	n.a.	n.a.	n.a.	6 (3.5)
Randomised	67 (100.0)	67 (100.0)	67 (100.0)	201 (100.0)
Safety set	67 (100.0)	67 (100.0)	67 (100.0)	201 (100.0)
Completed (PK Analysis set)	62 (92.5)	64 (95.5)	60 (89.6)	186 (92.5)
Withdrew before completion	5 (7.5)	3 (4.5)	7 (10.4)	15 (7.5)
Primary reason for withdrawal				
Unacceptable toxicity including AEs	4 (6.0)	3 (4.5)	6 (9.0)	13 (6.5)
Consent withdrawal by subject	0 (0.0)	0 (0.0)	1 (1.5)	1 (0.5)
Discretion of the Investigator	1 (1.5)	0 (0.0)	0 (0.0)	1 (0.5)
Related to COVID-19	4 (6.0)	3 (4.5)	6 (9.0)	13 (6.5)
Primary reason for withdrawal				
Unacceptable toxicity including AEs	4 (6.0)	3 (4.5)	6 (9.0)	13 (6.5)

Abbreviations:
AEs: adverse events; EU-UST: EU-sourced ustekinumab; N: number of subjects in the randomised set; n: number of subjects within assessment category; n.a.: not applicable; PK: pharmacokinetic; SB17: ustekinumab biosimilar candidate; US-UST: US-sourced ustekinumab.
Percentages were based on the number of randomised subjects.
Percentages of screening failure reasons were based on number of screening failures.

Supplementary table 2. Summary of PK parameters by post-dose ADAs status and treatment group

PK Parameter	Statistics	SB17 Positive N=18	SB17 Negative N=49
	n	18	44
AUC_{inf} (ng·h/mL)	Mean (SD)	4,882,600 (1,797,300)	5,250,400 (1,212,000)
	Median (Min-Max)	4,551,400 (2,414,000-8,884,000)	5,129,900 (2,723,000-8,834,000)
C_{max} (ng/mL)	Mean (SD)	5,179 (1,782)	5,061 (1,386)
	Median (Min-Max)	4,835 (2,630-8,730)	5,080 (2,730-9,030)
AUC_{last} (ng·h/mL)	Mean (SD)	4,583,900 (1,686,600)	4,777,100 (1,060,500)
	Median (Min-Max)	4,150,800 (2,302,000-8,526,000)	4,847,900 (2,610,000-7,733,000)
$AUC_{0\text{-}264h}$ (ng·h/mL)	Mean (SD)	1,044,900 (404,210)	1,044,700 (293,130)
	Median (Min-Max)	992,540 (458,500-1,780,000)	1,060,900 (470,000-1,673,000)
T_{max} (h)	Median (Min-Max)	264.000 (72-504)	168.000 (48-672)
V_z/F (mL)	Mean (SD)	6,633.9 (2,964.7)	7,940.3 (1,898.4)
	Median (Min-Max)	6,187.3 (2,817-12,910)	7,623.8 (5,146-12,420)
λ_z (1/h)	Mean (SD)	0.0019391 (0.0013913)	0.0011641 (0.00025457)
	Median (Min-Max)	0.0013989 (0.0008067-0.005569)	0.0011694 (0.0007264-0.001895)
$t_{1/2}$ (h)	Mean (SD)	484.82 (215.27)	622.75 (132.32)
	Median (Min-Max)	495.49 (124.5-859.2)	592.78 (365.8-954.2)
CL/F (mL/h)	Mean (SD)	10.387 (3.6284)	9.0397 (2.2178)
	Median (Min-Max)	9.8880 (5.065-18.64)	8.7722 (5.095-16.52)
$\%AUC_{extrap}$	Mean (SD)	5.94 (3.51)	8.71 (5.33)
	Median (Min-Max)	4.74 (1.4-14.6)	6.88 (2.4-30.2)

PK Parameter	Statistics	EU-UST Positive N=23	EU-UST Negative N=44
	n	22	42
AUC_{inf} (ng·h/mL)	Mean (SD)	4,455,800 (1,656,000)	5,273,000 (1,492,800)
	Median (Min-Max)	4,091,200 (2,045,000-8,627,000)	5,487,100 (3,167,000-10,280,000)
C_{max} (ng/mL)	Mean (SD)	5,374 (1,383)	5,854 (2,086)
	Median (Min-Max)	5,685 (2,950-7,290)	5,430 (3,260-13,700)
AUC_{last} (ng·h/mL)	Mean (SD)	4,176,500 (1,480,200)	5,208,200 (1,284,500)
	Median (Min-Max)	3,857,900 (1,974,000-7,752,000)	5,108,900 (3,012,000-9,786,000)
$AUC_{0\text{-}264h}$ (ng·h/mL)	Mean (SD)	1,141,400 (324,400)	1,151,700 (389,370)
	Median (Min-Max)	1,145,100 (601,400-1,660,000)	1,097,400 (565,500-2,820,000)
T_{max} (h)	Median (Min-Max)	120.000 (48.00-264.00)	168.000 (12.00-504.00)

V_z/F (mL)	Mean (SD)	6,898.5 (2,066.5)	7,280.7 (1,560.9)
	Median (Min-Max)	6,682.9 (3,117-11,560)	7,143.7 (2,825-11,290)
λ_z (1/h)	Mean (SD)	0.0018504 (0.0012854)	0.0011718 (0.00024946)
	Median (Min-Max)	0.0015573 (0.0009547-0.007060)	0.0011242 (0.0007386-0.001738)
$t_{1/2}$ (h)	Mean (SD)	459.81 (162.75)	618.27 (133.03)
	Median (Min-Max)	445.28 (98.2-726.0)	616.74 (398.7-938.4)
CL/F (mL/h)	Mean (SD)	11.509 (4.3848)	8.3858 (2.0669)
	Median (Min-Max)	10.999 (5.216-22.01)	8.2012 (4.378-14.21)
$\%AUC_{extrap}$	Mean (SD)	5.86 (2.74)	8.20 (3.95)
	Median (Min-Max)	5.15 (1.6-12.8)	7.37 (2.9-18.5)

PK Parameter	Statistics	US-UST Positive N=23	US-UST Negative N=44
	n	21	39
AUC_{inf} (ng·h/mL)	Mean (SD)	4,477,600 (1,030,800)	5,460,600 (1,647,100)
	Median (Min-Max)	4,498,200 (2,559,000-6,447,000)	5,438,500 (2,118,000-10,200,000)
C_{max} (ng/mL)	Mean (SD)	4,883 (1,317)	5,709 (1,766)
	Median (Min-Max)	4,790 (2,850-8,070)	5,660 (1,590-11,100)
AUC_{last} (ng·h/mL)	Mean (SD)	4,239,500 (956,640)	5,055,500 (1,433,100)
	Median (Min-Max)	3,990,900 (2,434,000-6,019,000)	5,133,000 (1,959,000-9,257,000)
$AUC_{0\text{-}264h}$ (ng·h/mL)	Mean (SD)	1,024,700 (317,540)	1,167,700 (369,000)
	Median (Min-Max)	946,320 (482,900-1,704,000)	1,190,300 (291,500-2,042,000)
T_{max} (h)	Median (Min-Max)	168(48-1,008)	168.000 (48-384)
V_z/F (mL)	Mean (SD)	7,163.6 (2,211.3)	7,282.0 (2,285.2)
	Median (Min-Max)	6,932.0 (3,207-12,520)	6,001.6 (3,624-17,980)
λ_z (1/h)	Mean (SD)	0.0015615 (0.00040252)	0.0012552 (0.00027122)
	Median (Min-Max)	0.0014812 (0.0007192-0.002264)	0.0012276 (0.0008110-0.002108)
$t_{1/2}$ (h)	Mean (SD)	477.13 (147.58)	575.50 (115.46)
	Median (Min-Max)	467.96 (306.1-963.8)	564.64 (328.8-854.7)
CL/F (mL/h)	Mean (SD)	10.601 (2.6252)	9.0725 (3.1883)
	Median (Min-Max)	10.004 (6.980-17.59)	8.2744 (4.411-21.25)
$\%AUC_{extrap}$	Mean (SD)	5.16 (3.28)	6.95 (2.98)
	Median (Min-Max)	4.10 (2.2-13.8)	6.01 (2.7-14.9)

Abbreviations:
ADAs: anti-drug antibodies; $AUC_{0\text{-}264h}$: AUC from time zero to 264 hours; AUC_{last}: AUC from time zero to the last quantifiable concentration; AUC_{inf}: area under the concentration-time curve from time zero to infinity; C_{max}: maximum serum concentration; CL/F: apparent clearance; EU-UST: EU-sourced ustekinumab; Min: minimum; Max: maximum; N: number of subjects in the PK analysis set with the corresponding post-dose ADA result; n: Number of subjects for the assessment parameter; PK: pharmacokinetic; SB17: ustekinumab biosimilar candidate; SD: standard deviation; $t_{1/2}$: terminal half-life; T_{max}: time to reach C_{max}; US-UST:

US-sourced ustekinumab; V_z/F: apparent volume of distribution during the terminal phase; λ_z: terminal rate constant; $\%AUC_{extrap}$: percentage of AUC_{inf} due to extrapolation from time of last measurable concentration to infinity.
Median and Min-Max range were summarised for T_{max}.
Samples with low-speed centrifuge issue were excluded from PK parameters calculation. Refer to Pharmacokinetic evaluation of Results section.
